# Longitudinal treatment patterns in myasthenia gravis: An analysis from the prospective Dutch-Belgian patient registry

**DOI:** 10.1177/22143602251405924

**Published:** 2025-12-09

**Authors:** Linda Remijn-Nelissen, Martijn R Tannemaat, Jan JGM Verschuuren

**Affiliations:** 1 Department of Neurology, 4501 Leiden University Medical Center, Leiden, the Netherlands

**Keywords:** myasthenia gravis, treatment, pyridostigmine, corticosteroids, immunosuppressants, intravenous immunoglobulin therapy

## Abstract

**Background:**

Understanding treatment patterns in myasthenia gravis (MG) is crucial for clinical practice. However, longitudinal data are limited.

**Objective:**

This study aimed to provide an overview of treatment patterns in different subgroups throughout the disease course.

**Methods:**

All adult patients with acetylcholine receptor (AChR), muscle-specific kinase (MuSK), or seronegative MG enrolled in the Dutch-Belgian myasthenia registry were included. AChR-MG patients were classified into early-onset (EOMG) (≤50 years) and late-onset (LOMG) (>50 years) MG. Mixed-effects regression was used to compare differences between the different subgroups.

**Results:**

A total of 506 patients were included (147 AChR EOMG, 265 AChR LOMG, 26 MuSK, and 94 seronegative MG patients). Data were available within a year of diagnosis for 84 patients. Most patients initially started treatment with pyridostigmine (93%; 78/84), followed by corticosteroids (52%; 44/84) and azathioprine or mycophenolate mofetil (30%; 25/84). After ten years, the use of pyridostigmine (65%; 54/83) and corticosteroid use (36%; 30/83) declined, whereas the use of azathioprine and mycophenolate mofetil increased (55%; 46/83). Seronegative patients received fewer treatments than AChR MG patients, independent of functional status and disease duration (OR 0.38, 95% CI 0.17–0.84, p = 0.018), and were less likely to use corticosteroids (OR 0.15, 95% CI [0.05–0.41], p < 0.001). Patients with LOMG reported more frequently immunosuppressive use compared to patients with EOMG (OR 4.69, 95% CI 1.16–19.02, p = 0.031).

**Conclusions:**

This analysis offers valuable insights into treatment patterns in different subgroups of patients with MG. A substantial proportion of patients continues to rely on corticosteroids, suggesting an ongoing need for alternative treatment options.

## Introduction

Myasthenia gravis (MG) is a rare, chronic autoimmune disorder characterized by muscle fatigability resulting from autoantibodies aimed at components of the neuromuscular junction.^1^ Various studies have identified distinct subgroups of MG, primarily based on antibody status and clinical features.^2^ Patients may present with antibodies directed against the acetylcholine receptor (AChR), muscle-specific kinase (MuSK), or may be seronegative, demonstrating no detectable antibodies. In addition to serological classification, disease characteristics have been shown to differ across age groups.^2^ Early-onset MG (EOMG), defined as disease onset before the age of 50, predominantly affects young women and is commonly associated with AChR antibodies, thymic hyperplasia, and the HLA-A1-B8-DR3 haplotype. In contrast, late-onset MG (LOMG), which manifests after the age of 50, occurs more frequently in men and is associated with thymic atrophy and the HLA-DRB1*15:01 allele.^3^

The treatment goal for MG is achieving Minimal Manifestation Status or better, with no functional limitations and only mild medication side effects.^4^ This often involves a sequential approach, requiring trial and error to identify the most effective regimen for each patient. First-line pharmacological treatment for MG consists of acetylcholinesterase inhibitors, which enhance neuromuscular transmission by increasing the amount of acetylcholine in the synaptic cleft. Although various acetylcholinesterase inhibitors exist, pyridostigmine is the preferred agent for symptomatic treatment in clinical practice.^4^ If symptom control proves insufficient with pyridostigmine alone, corticosteroids are introduced, often in combination with non-steroidal immunosuppressive agents. Corticosteroids play a central role in MG treatment due to their faster onset of action compared to other immunosuppressive therapies, affordability, and wide availability.^5^ Nevertheless, long term corticosteroid use is known to be associated with significant adverse effects.^6^ Non-steroidal immunosuppressant therapies (NSISTs), such as azathioprine, mycophenolate mofetil, ciclosporin, and rituximab, can be added to the treatment regimen to reduce the cumulative corticosteroid dose. However, due to lack of comparative studies, there is considerable global variation in the preferred use of these therapies.^7^ For patients experiencing acute exacerbations, intravenous immunoglobulin therapy (IVIg) or plasma exchange (PE) is used. Additionally, IVIg may be considered as a long-term treatment option in patients in whom prior therapeutic interventions fail to achieve adequate disease control, although a recent trial did not demonstrate the efficacy of this strategy.^8^

In recent years, significant advances have been made in the treatment of MG with the introduction of complement inhibitors and FcRn blockers. At present, in most countries, these new treatments are reserved for patients with severe, refractory disease, partly due to their high costs. However, the definition of refractory MG is not clearly established. In general, refractory disease refers to the failure of multiple prior therapies, and it is common practice that several other treatments must first be attempted before initiating these new agents. Therefore, in order to estimate who may benefit from emerging treatments, it is essential to understand how current therapies, including pyridostigmine, corticosteroids, NSISTs, IVIg, and PE, are used throughout the disease course. These insights are key to refining treatment guidelines and optimizing therapeutic strategies for patients with MG. This study therefore aims to provide a comprehensive overview of longitudinal treatment patterns of currently available treatment options in different subgroups of MG patients.

## Patients and methods

### Data source

We used data from the Dutch-Belgian registry for neuromuscular junction disorders, a prospective clinical registry in the Netherlands and Belgium, with the primary aim to study the natural disease history, treatment response, and potential risk factors. Full details of the design of the registry, along with the primary results, have been reported previously.^9^ In short, the registry is a collaborative initiative of the Dutch patient support organization for neuromuscular diseases and Leiden University Medical Center, a tertiary center for the treatment of neuromuscular junction disorders. The registry is an ongoing longitudinal database which collects medical information obtained from both patients and their treating physicians. The study was reviewed and approved by the independent Medical Ethics Review Committee Leiden The Hague Delft. All patients provided written informed consent before participation in the registry.

### Outcomes

In this analysis, we included data from all patients with a confirmed diagnosis of MG, collected from August 2016 to March 2025, including those with disease onset at ≥18 years of age.

Diagnosis was based on clinical signs or symptoms suggestive of auto-immune MG, along with a positive serologic test for AChR antibodies or MuSK antibodies, or a diagnostic electrophysiological investigation consistent with a neuromuscular transmission disorder. Registry participants with congenital myasthenic syndrome or Lambert-Eaton myasthenic syndrome were excluded.

Data collected at baseline included patient demographics, disease characteristics such as antibody status, date of diagnosis, thymectomy (yes/no), and functional status, as well as treatment characteristics, including the use of the following treatments either at the time of data collection or within the past three months: pyridostigmine, prednisolone, azathioprine, mycophenolate mofetil, rituximab, ciclosporin, methotrexate, cyclophosphamide, IVIg, or PE. In addition, patients were asked whether they had ever been treated with IVIg or PE at any point during the course of their disease, and whether they had ever had to discontinue treatments for MG due to side effects.

During each scheduled annual follow-up questionnaire, patients were again asked about their functional status, treatments they were receiving either at the time of data collection or within the past three months, as well as whether they had ever had to discontinue treatments for MG due to side effects.

Seronegative patients were defined as patients who had no detectable antibodies before or at the time of enrollment in the registry. Early-onset MG (EOMG) was defined as an age of onset of 50 years or younger, and late-onset MG (LOMG) as an age of onset greater than 50 years. Data are presented by disease duration, with each duration representing a prevalent patient group. While there is overlap between groups, not all patients appear in every duration group.

### Statistical analysis

Mean and standard deviation (SD), or median and interquartile range [IQR] were used to report continuous variables where appropriate. Comparisons were based on either student's T-test (normal distribution) or Mann-Whitney U test (non-normal distribution). Binary and categorical variables were reported according to frequencies and percentages. Categorical data are presented as proportions (%) and comparisons were assessed by the Chi-Square test. P-values were considered significant when <0.05. Missing data were reported and excluded from the analyses.

To investigate the association between treatment characteristics and disease characteristics, we applied several mixed-effects regression models. For ordinal outcomes, such as the number of treatments used over the course of the disease and the functional status of patients, mixed-effects ordinal regression was used. In the first model, we examined how the number of treatments varied in relation to disease onset or antibody subtype, disease duration, and functional status, including patients as a random effect. In the second model, functional status was analyzed as the outcome variable, with disease onset or antibody subtype and disease duration as fixed effects, and again, a random intercept for patients. For binary outcomes, including the use of specific treatment modalities such as pyridostigmine, corticosteroids, NSISTs, and IVIg/PE, and side effects, we employed mixed-effects logistic regression models. Each model assessed the association between treatment use or side effects and disease onset or antibody subtype, adjusting for disease duration and functional status, while accounting for repeated measures by including patients as a random effect. All data analyses were conducted using RStudio (version 4·4.0)

## Results

Overall, 506 patients with MG enrolled in the Dutch-Belgian MG registry were included in this analysis (mean age 61 [SD 14]), of whom 247 (49%) were female. The cohort consisted of 412 patient with AChR MG, of whom 147 patients with EOMG and 265 patients with LOMG, 26 patients with MuSK MG, and 94 patients with seronegative MG. The median number of annual observations was 4 (range 1, 8). Patient demographics and disease characteristics, stratified by the different subgroups are summarized in Table 1.

**Table 1.** Baseline characteristics.

	Missing, n (%)	Overall, n = 506	AChR EOMG, n = 147	AChR LOMG, n = 265	MuSK, n = 26	Seronegative, n = 94
Age at diagnosis	0 (0%)	53 (16)	36 (10)	64 (8)	49 (14)	51 (13)
Age at enrollment in registry	0 (0%)	61 (14)	49 (14)	68 (8)	57 (13)	57 (12)
Gender, female	0 (0%)	247 (49%)	105 (71%)	85 (32%)	18 (69%)	57 (61%)
Disease duration, median (IQR)	0 (0%)	4 (1, 11)	11 (4, 21)	3 (1, 6)	5 (1, 12)	3 (0, 13)
BMI	17 (3.4%)	27.4 (5.4)	26.2 (5.8)	28.0 (5.0)	27.4 (4.9)	27.5 (5.5)
Thymectomy, yes	211 (42%)	97 (33%)	64 (67%)	29 (18%)	3 (20%)	4 (9.8%)
Symptoms at baseline, generalized	16 (3.0%)	482 (93%)	141 (97%)	239 (93%)	26 (100%)	76 (85%)
Functional status	4 (0.8%)					
Fully active, no limitations		153 (30%)	46 (32%)	78 (30%)	5 (19%)	29 (31%)
Fully dependent, entirely bed or chair-bound		4 (0.8%)	0 (0%)	3 (1.1%)	0 (0%)	1 (1.1%)
Limited self-care, mostly bed or chair-bound		17 (3.4%)	5 (3.4%)	7 (2.7%)	1 (3.8%)	5 (5.3%)
Restricted heavy activity, light work capable		230 (46%)	63 (43%)	131 (50%)	15 (58%)	36 (38%)
Self-care possible, unable to work, mostly out of bed		98 (20%)	32 (22%)	43 (16%)	5 (19%)	23 (24%)

Numbers are mean (SD) or n (%) unless otherwise specified. AChR: acetylcholine receptor; EOMG: early-onset myasthenia gravis; LOMG: late-onset myasthenia gravis; MuSK: muscle-specific kinase; BMI: body mass index.

Data were available for 84 patients within the first year after diagnosis. During this period, nearly all patients (99%; 83/84) initiated treatment. Most started with pyridostigmine (93%; 78/84), over half began corticosteroids (52%; 44/84), and nearly one-third started azathioprine or mycophenolate mofetil (30%; 25/84). Corticosteroids or other NSISTs were not used in 38 patients. While the difference was not statistically significant, there was a tendency for these patients to be more frequently female (55%; 21/38) than those who did receive such treatments (35%; 16/46), p = 0.060. No difference was found in age at diagnosis, thymectomy, symptoms at baseline (ocular vs. generalized), or functional status.

At the five-year mark, treatment patterns shifted somewhat: 63% (107/170) of patients remained on pyridostigmine, 44% (74/170) continued corticosteroids, and the use of azathioprine or mycophenolate mofetil increased to 46% (79/170). After ten years, a majority (65%; 54/83) were still using pyridostigmine, corticosteroid use declined to 36% (30/83), while more than half (55%; 46/83) received treatment with azathioprine or mycophenolate mofetil. As the disease duration increased, the number of treatments decreased (OR 0.85, 95% CI [0.80–0.90], p < 0.001).

**Figure 1.**
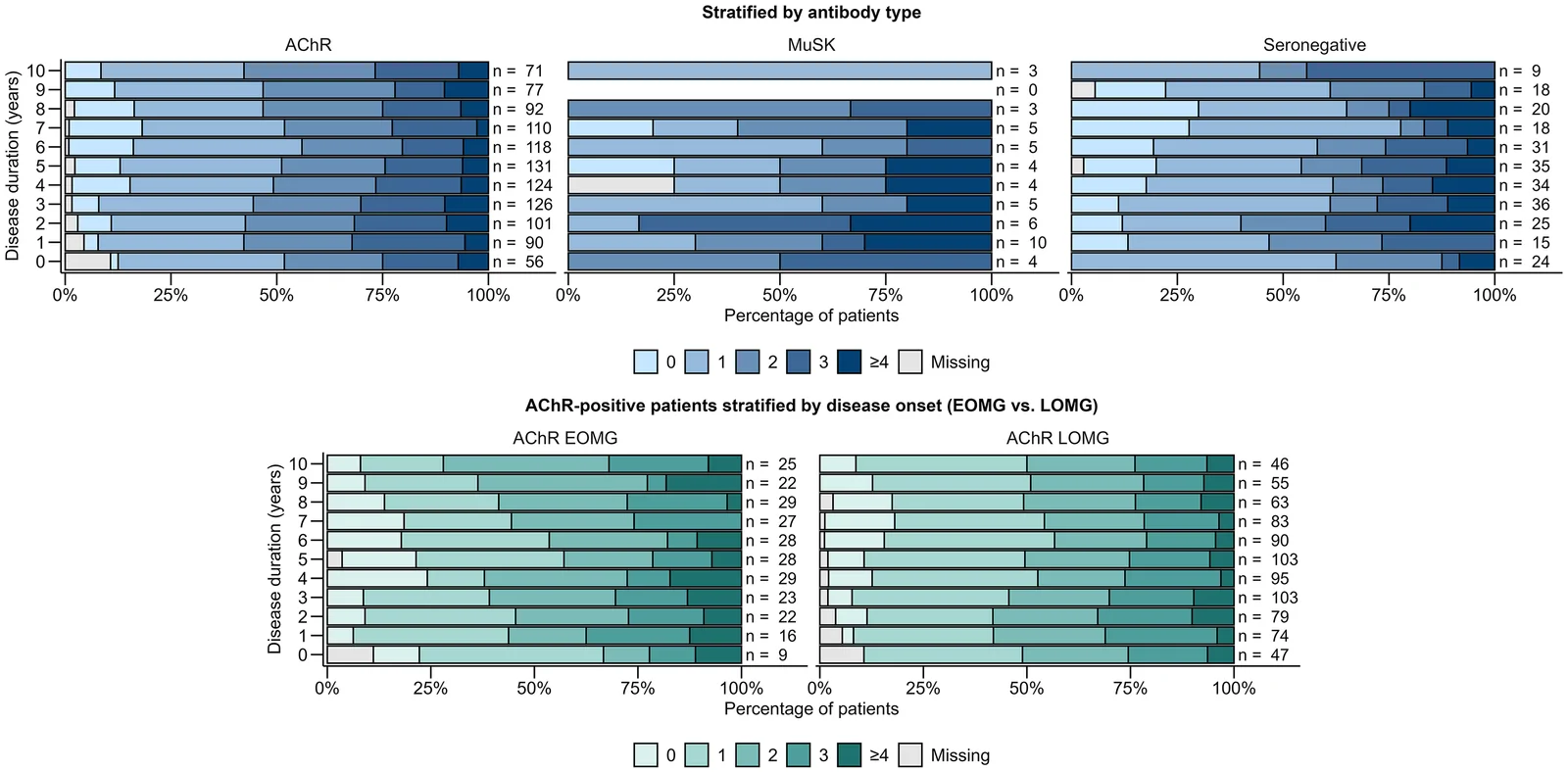
Number of concurrent treatments by disease duration in different subgroups of MG patients. Each bar represents a prevalent patient group at a certain disease duration, and patients in one disease duration group may not necessarily be present in other duration groups. Treatments include pyridostigmine, corticosteroids, non-steroidal immunosuppressant therapies, intravenous immunoglobulin therapy, and plasma exchange. Abbreviations: AChR: acetylcholine receptor; MuSK: muscle-specific kinase; EOMG: early-onset myasthenia gravis; LOMG: late-onset myasthenia gravis.

In Figure 1, the number of treatments used by patients within the first ten years of the disease is shown, stratified by the different subgroups. Seronegative patients received fewer treatments, independent of functional status and disease duration, compared to AChR MG patients (OR 0.38, 95% CI 0.17–0.84, p = 0.018). No association was found between disease onset (EOMG vs. LOMG) and the number of treatments after adjusting for disease duration and functional status (OR 0.69, 95% CI [0.29–1.67], p = 0.414), nor was there an association between thymectomy (yes vs. no) in AChR-MG and the number of treatments (OR 1.41, 95% CI [0.75–2.65], p = 0.281).

**Figure 2.**
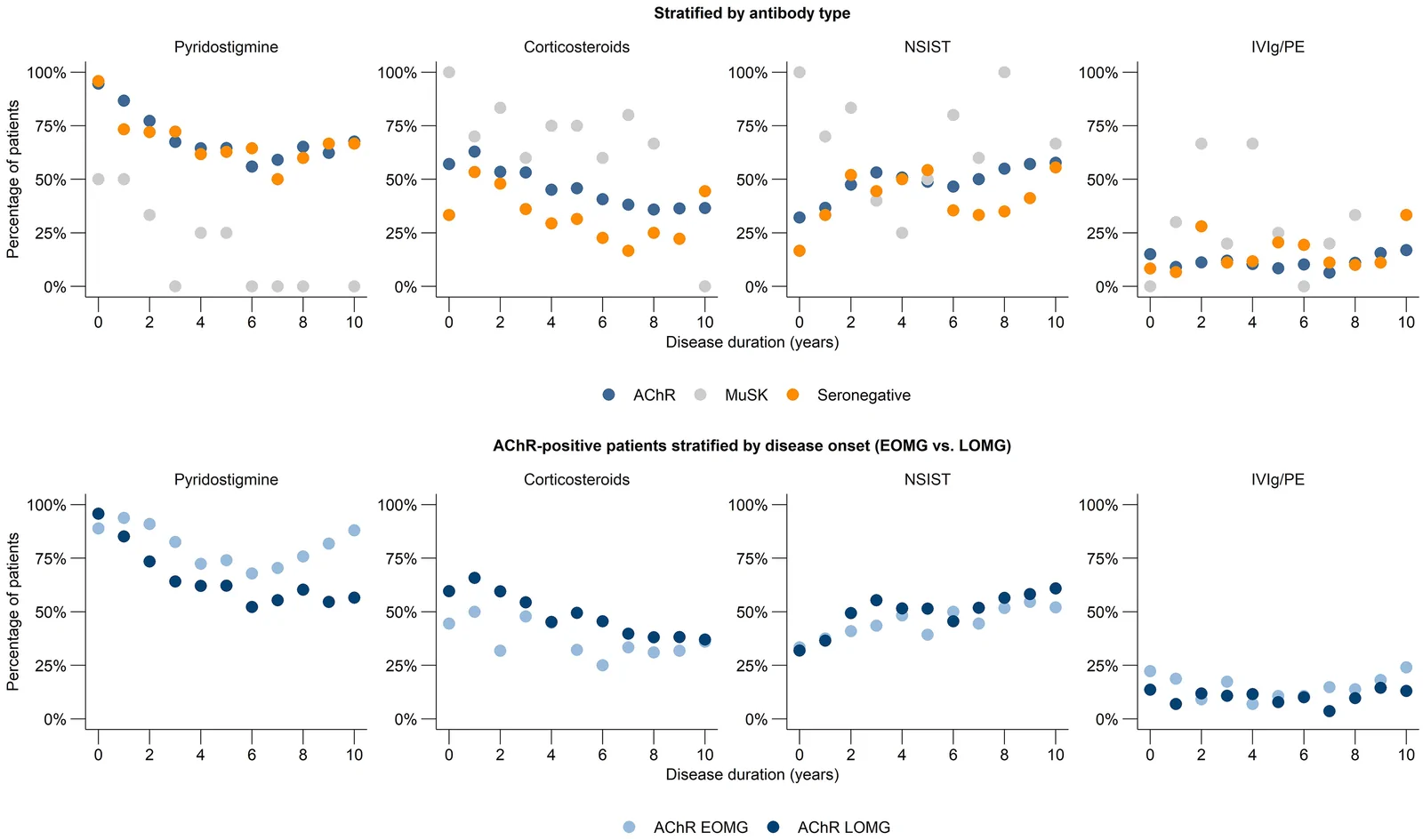
Evolution of treatment modalities throughout the disease course. AChR: acetylcholine receptor; MuSK: muscle-specific kinase; EOMG: early-onset myasthenia gravis; LOMG: late-onset myasthenia gravis; NSIST: non-steroidal immunosuppressant therapies (azathioprine, mycophenolate mofetil, ciclosporin, methotrexate, rituximab, cyclophosphamide); IVIg: intravenous immunoglobulin therapy; PE: plasma exchange.

Figure 2 shows the evolution of different treatment modalities (pyridostigmine, corticosteroids, NSIST, and IVIg/PE) throughout the disease course, stratified for the different subgroups. Pyridostigmine was the most commonly used treatment in all subgroups except MuSK MG, with its use declining as the disease duration increased (OR 0.53, 95% CI [4.62–0.61], p < 0.001). Patients with LOMG reported less frequent use of pyridostigmine compared to patients with EOMG in the first ten years of the disease, adjusted for disease duration and functional status (OR 0.05 95% CI 0.01–3.04, p = 0.001). Additionally, patients who underwent a thymectomy used pyridostigmine more frequently than those who did not (OR 8.54, 95% CI [1.75–41.55], p = 0.008). Corticosteroid use declined with a longer disease duration, independent of functional status (OR 0.79, 95% CI [0.73–0.85], p < 0.001). Seronegative patients used corticosteroids less frequently compared to AChR MG patients (OR 0.15, 95% CI [0.05–0.41], p < 0.001). A trend was observed, although not statistically significant, suggesting that patients with EOMG used corticosteroids less frequently compared to those with LOMG (OR 2.53, 95% CI [0.91–7.09], p = 0.077), after accounting for differences in disease duration and functional status. Corticosteroid use did not differ between patients who underwent a thymectomy and those who did not (OR 0.67, 95% CI [0.26–1.72], p = 0.400). The use of NSISTs increased with a longer disease duration across all patients (OR 1.18, 95% CI [1.09–1.28], p < 0.001). MuSK MG patients appeared to use NSISTs more frequently than AChR MG patients (OR 14.70, 95% CI [1.64–131.97], p = 0.016), while there was a trend, although not statistically significant, suggesting that seronegative patients used NSISTs less frequently than those with AChR MG (OR 0.32, 95% CI [0.10–1.06], p = 0.063). The use of NSISTs did not differ between patients who underwent a thymectomy and those who did not (OR 1.42, 95% CI [0.60–3.36], p = 0.428).

Patients with LOMG reported more frequent use of immunosuppressive therapies in general (corticosteroids, either alone or in combination with a NSIST) compared to patients with EOMG (OR 4.69, 95% CI 1.16–19.02, p = 0.031). The use of immunosuppressive therapies was comparable between female and male patients (OR 2.35, 95% CI [0.68–8.17], p = 0.179).

There was no difference in frequency of use of IVIg/PE between the different subgroups. Out of all patients, 33.6% (179 patients) reported to have ever used IVIg during the disease course, and 16.4% (87 patients) reported to have ever been treated with PE. After adjusting for functional status, a longer disease duration was associated with a reduced likelihood of receiving plasma exchange (OR 0.78, 95% CI [0.63–0.95], p = 0.014), while it was not associated with the likelihood of receiving intravenous immunoglobulin (OR 0.97, 95% CI [0.94–1.01], p = 0.167). No significant differences in the likelihood of receiving plasma exchange or IVIg were observed between antibody subtypes. However, there was a trend that patients with LOMG were less likely to receive IVIg (OR 0.32, 95% CI [0.11–0.96], p = 0.043) and plasma exchange (OR 0.17, 95% CI [0.03–1.11], p = 0.064).

Treatment discontinuation due to side effects was most frequently reported in in the first year after diagnosis: 19/84 patients (23%) discontinued treatment due to side effects. After five years, this number decreased to 20/170 patients (12%), and after ten years, 9/83 patients (11%). The likelihood of experiencing side effects decreased throughout the first ten years of the disease course (OR 0.87, 95% CI [0.81–0.94], p < 0.001). There was no difference between antibody subtype, or EOMG and LOMG.

**Figure 3.**
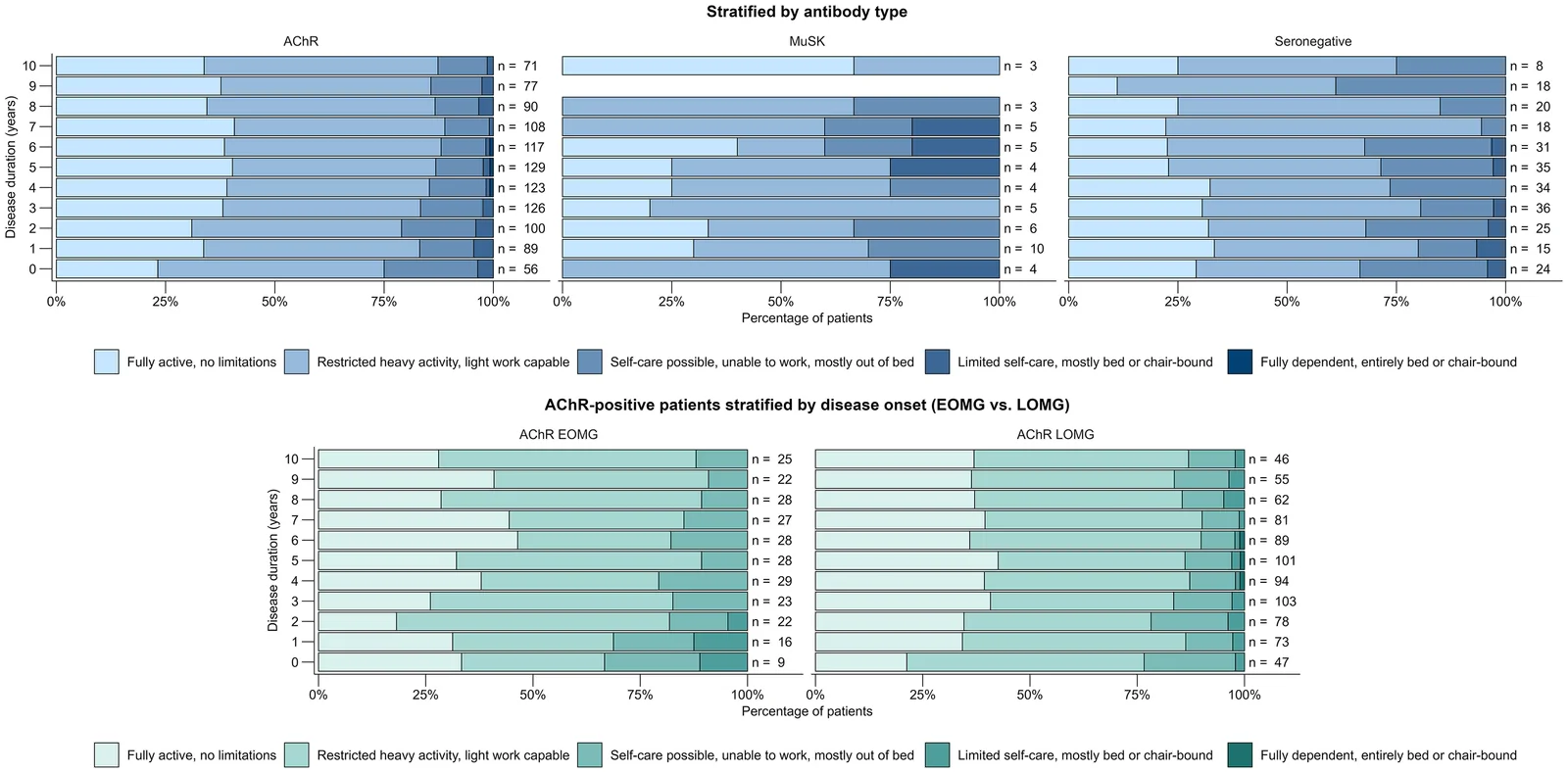
Functional status by disease duration in different subgroups of MG patients. AChR: acetylcholine receptor; MuSK: muscle-specific kinase; EOMG: early-onset myasthenia gravis; LOMG: late-onset myasthenia gravis.

In Figure 3 the evolution of the functional status throughout the first ten years of the disease course is shown, stratified for the different subgroups. There appears to be a trend indicating that seronegative patients have a worse functional status compared to AChR MG patients, after adjusting for disease duration (OR 2.40, 95% CI [0.96–6.00], p = 0.061). No difference was found between functional status between EOMG and LOMG.

## Discussion

The current study provides an overview of longitudinal characteristics and treatment patterns of currently available pharmacological treatment options in a large cohort of MG patients. This cohort describes a time period in which complement inhibitors and FcRn blockers were not yet widely prescribed for patients with MG in the Netherlands, enabling the identification of unmet medical needs and potential therapeutic opportunities for emerging treatments. The observed treatment patterns in this study align well with our experiences in clinical practice; most patients require pharmacological treatment for MG; the majority of patients are initially treated with pyridostigmine, but this proportion subsequently declines to levels similar to those previously reported;^10^ the use of corticosteroids declines after the first two years, while the use of NSIST increases during the disease course.

Our analysis shows that ten years after disease onset, 30–40% of patients continue to rely on corticosteroids, although the national guideline emphasizes the need for efforts to minimize their use. These findings are supported by previous studies that, while describing a shorter disease duration of 3 and 5 years, report similar percentages of patients using corticosteroids (42%,^11^ and 45%,^12^ respectively). Although, based on our data, we cannot differentiate between patients who use corticosteroids chronically and those requiring (short-term) corticosteroids to manage disease fluctuations, these findings suggest an ongoing need for alternative treatment options.

In this study, we described treatment patterns among seronegative MG patients. Since antibody status is only assessed at the time of registry inclusion, it cannot be ruled out that some of these patients may have tested positive for antibodies later in the disease course. Nonetheless, we demonstrate that this group received fewer treatments overall and was less frequently treated with immunosuppressive therapies. Interestingly, they did not appear to receive IVIg or plasma exchange less often.

In addition, it is notable that patients with LOMG more frequently received immunosuppressive treatment compared to those with EOMG, despite EOMG being associated with a more refractory disease course.^13,14^ One possible explanation is that patients with EOMG more often require treatments such as IVIg and plasma exchange, thereby reducing the need for long-term immunosuppressive therapies.

During the first ten years after disease onset, 10–15% of patients reported treatment with IVIg annually, and a smaller proportion (<3%) of patients reported treatment with PE. The proportion of IVIg use reported in this study is higher than previously described; earlier studies have reported proportions ranging between 2% and 10%.^12,15,16^ Several factors may explain this difference. More severe patients may have been overrepresented in our cohort due to the voluntary nature of the registry and the fact that patients treated at the national expert center, who are often more complex cases, are more likely to be encouraged to join the registry than those treated in other hospitals. Moreover, in the Netherlands, IVIg is easily accessible, with the option for home administration, and it is fully reimbursed under the basic health insurance package, which may contribute to its frequent use.

This study has several limitations. First, treatment data in the registry are patient-reported, making them susceptible to subjectivity and recall bias. For example, patients may have misunderstood the question regarding treatment use at the time of data collection or within the past three months, and may have reported treatments they had ever used instead. Furthermore, although cyclophosphamide is queried in the registry, to our knowledge, it is not prescribed in the Netherlands for this indication. It is possible that patients may have confused it with ciclosporin, or that they were treated with cyclophosphamide for other indications. Second, information on medication dosages is not collected, limiting conclusions regarding treatment intensity. Third, treatment data are recorded annually and reflect therapies received either at the time of reporting or in the preceding three months. As a result, short-term treatments such as corticosteroids, IVIg, or PE administered in the preceding nine months may not have been captured. Fourth, the number of patients with MuSK MG was limited in this cohort. Therefore, the results for this subgroup should be interpreted with caution. Fifth, validated outcome measures (such as the MG-ADL) are unfortunately not collected in the registry. Therefore, we chose to use the question regarding functional outcome as a proxy for disease severity. As this is not a validated measure, it limits the interpretability of our findings. Sixth, data on thymectomy were missing in a substantial number of patients (44%), which may have affected the reliability of related analyses. Finally, the vast majority of patients have been treated in the Netherlands; differences in healthcare systems and reimbursement policies in other countries may influence treatment practices and limit generalizability. Despite these limitations, we believe that this real-world analysis provides important insights into treatment patterns throughout the disease course in patients with MG.

In conclusion, in this study we demonstrate that a substantial proportion of patients continues to rely on corticosteroids or IVIg/PE throughout the course of the disease. These findings suggest an ongoing need for alternative therapeutic options. The introduction of novel therapies may help to address these challenges, but their long-term impact on treatment patterns will need to be evaluated in future research.
